# Photochemistry and haze in chemically reducing terrestrial atmospheres

**DOI:** 10.1016/j.fmre.2026.04.008

**Published:** 2026-04-13

**Authors:** Lixiang Gu, Siteng Fan

**Affiliations:** Department of Earth and Space Sciences, Southern University of Science and Technology, Shenzhen 518055, China

**Keywords:** Terrestrial planets, Atmospheric chemistry, Photochemical haze, Planetary habitability, Atmospheric evolution

## Abstract

Properties and processes within planetary atmospheres are critical for surface habitability. Evidence from the Earth suggests that our only known form of life has likely originated in a chemically reducing atmosphere. The photochemistry and haze formation in such environments played an important role in the emergence and sustenance of early life, yet these processes have received insufficient attention due to reliance on outdated laboratory measurements and numerical simulations. This perspective aims to highlight the importance of chemically reducing terrestrial atmospheres, elucidate the key chemical and physical processes involved, and propose integrated experimental and modeling strategies to overcome current limitations.

## 1 Introduction

The atmosphere of a terrestrial planet plays a pivotal role in regulating its surface environment, serving as an interface between the planetary interior and outer space. After the fast escape of the primordial atmosphere, the composition and properties of a secondary atmosphere, where the Earth’s life has emerged, were primarily determined by interior outgassing and impact delivery during the early ages, which are further modulated by processes during the evolution of the planet [[1], [2], [3]]. These processes include surface weathering, exchange with a possible surface ocean, solar radiation, atmospheric escape, etc. [1,2]. While substantial studies have focused on Earth’s present-day oxidizing atmosphere, our understanding of chemically reducing atmospheres, which were likely present on the prebiotic and early Earth, remains limited. Nevertheless, scenarios of the origin of life usually invoke reducing environments, as such conditions favor the synthesis and survival of key life-building blocks, including amino acids, nucleobases, and simple sugars [4,5]. Investigations of Solar System bodies that have present-day reducing terrestrial atmospheres, e.g., Titan, Triton, and Pluto, agree with these scenarios, where rich photochemistry and haze formation are present, with productions of complex organics [6,7]. Therefore, studying the processes in chemically reducing atmospheres holds broad significance for understanding planetary habitability in general.

This perspective focuses on the photochemistry, haze microphysics, and radiative-chemical processes in chemically reducing terrestrial atmospheres. Our emphasis is on the early Earth’s atmosphere, but it is also relevant to other terrestrial atmospheres and exoplanets. In this perspective, we first provide an overview of current knowledge on the properties and processes in Earth’s prebiotic atmosphere in Section 2. Section 3 presents the major approaches through which this understanding has been developed, as well as their current limitations. We then propose potential solutions in Section 4.

## 2 Prebiotic atmosphere on Earth

Earth is the only planet known to support life. The prebiotic atmosphere on the early Earth offers the sole evidence of an environment where life has emerged. As a secondary atmosphere, its initial composition was largely determined by the redox state of the mantle and impact delivery during the first several 100 million years after the planetary formation, and then continually modulated by atmospheric escape [1,3]. Though debates persist regarding the prebiotic redox state of this atmosphere, studies on the origin of life usually prefer a reducing environment [3,4]. Geochemical proxies also indicate that a low oxygen condition was present on Earth at the time life emerged (e.g., [1]), which was maintained throughout the Archean Eon until the Great Oxidation Event.

In such a reducing atmosphere, gases such as H₂, CH₄, NH₃, and H₂S are proposed to be abundant (Fig. 1; [1,2]). Though the fluxes of these gases are still under exploration, the mass-independent sulfur isotope anomalies (S-MIF) require a CH_4_ abundance >20 ppmv, 10 times the present-day value [1], which is likely accompanied by high concentrations of H₂, NH₃, and H₂S. Photochemical reactions involving these molecules could have potentially produced prebiotic compounds, as suggested by the nucleobases and amino acids observed in both laboratory experiments and models [4,5]. Additionally, these gases may also contribute to resolving the faint young Sun paradox in addition to the elevated CO_2_ (e.g., [8]) through the greenhouse effect. Moreover, collision-induced absorption between gas pairs of N_2_—H_2_ and CO_2_—H_2_ can broaden the absorption spectra, which further enhances the greenhouse effect and warms the planet even with a minor H_2_ constituent [9]. All these processes combined have likely contributed to maintaining the liquid surface water on the early Earth at the time when the Sun was only about 75% as bright as it is today.

**Fig. 1:**
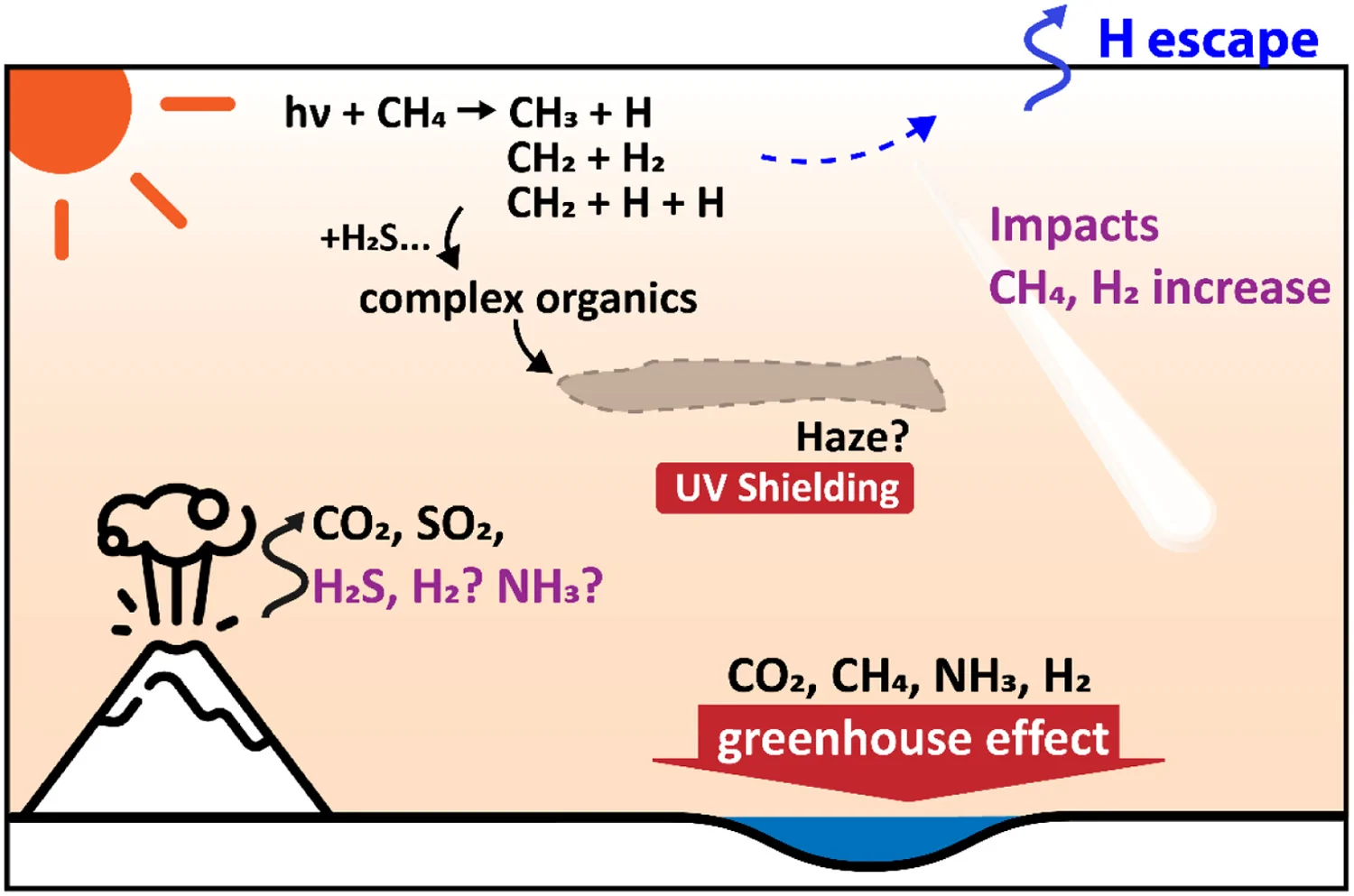
**Illustration of possible processes in reducing atmospheres on the early Earth**. Texts in purple denote the processes that help create or maintain the reducing environment, while those in blue represent the oxidizing processes. The red arrow and rectangle denote the radiative effects.

Given a reducing atmosphere enriched in H₂, CH₄, NH₃, organic hazes are expected to form through photochemistry. Though the very existence of enduring organic hazes is still under debate (Fig. 1), field-based proxies, e.g., the coupled sulfur and carbon isotopic anomalies, suggest episodic and regional haze formation in the Neoarchean [10]. Impacts in the early ages can also lead to, possibly global, haze layers [3]. These haze particles could have significant radiative and climate impacts, influencing the atmospheric temperature and the planetary energy budget, similar to those observed on present-day icy satellites [6,7]. They may also have offered UV shielding on the early Earth that helped preserve the reducing gases below and possibly protected the early life on the planetary surface (Fig. 1; e.g., [1]). The macromolecular haze particles could also serve as condensation nuclei to create microenvironments in airborne droplets enriched in dissolved organics, which could provide localized niches with high concentrations of prebiotic compounds and reactants, leading to the rainout of prebiotic molecules and potentially facilitating prebiotic synthesis [11]. This highlights the potentially significant role of haze in planetary habitability and prebiotic synthesis.

Episodic and catastrophic events, such as volcanic outgassing and meteorite impacts, have likely contributed to producing and maintaining reducing environments and/or haze formation. However, the detailed influence of such processes on atmospheric composition is currently poorly understood with large uncertainties (Fig. 1). Laboratory experiments suggest that sulfur-bearing gases from outgassing may have significantly catalyzed organic polymerization [12], leading to haze formation under lower CH₄/CO₂ ratios than previously thought in CHNO atmospheres. Moreover, impact-induced chemistry may have generated pulses of H₂ and CH₄, creating transient reducing conditions lasting up to tens of millions of years [3]. Both processes were far more frequent in the early ages than today, when both the planet and the Solar System were highly dynamically active.

In summary, the secondary atmosphere of the prebiotic Earth is potentially chemically reducing. Abundant reducing gases have likely contributed to the production of prebiotic compounds and to keeping the planet sufficiently warm. Photochemical hazes would have formed, further modulating the atmospheric properties and creating airborne microenvironments. Episodic outgassing and impacts have been occasionally and dynamically altering such an environment. All these coupled processes are significant but currently with large uncertainties, which require efforts from multiple disciplines.

## 3 Approaches and limitations

The study of photochemistry and haze formation in the Earth’s early atmosphere falls within the field of planetary atmospheric science, given its substantial differences from the present day. Numerical models and laboratory experiments developed for planetary conditions have been widely adapted in advancing our understanding. However, limitations have arisen recently with the increasing observational/geochemical constraints.

### 3.1 Numerical models

Current numerical models developed for the Hadean and Archean atmospheres are based on those originally designed for Titan [13]. These models have greatly advanced our understanding in the past few decades, yet they typically assume the haze optical and microphysical properties to be identical to those of tholins, the laboratory analog of Titan’s haze. This assumption has never been validated and is therefore likely questionable, since the haze compositional and morphological properties can vary substantially across planets, as evidenced by Pluto observations [6]. Moreover, the modeled vertical transport and sedimentation processes are commonly simplified through one-dimensional eddy diffusion and settling parameterizations with atmospheric dynamics neglected (e.g., [13]). Some efforts have been made with 3D general circulation models [14], but the haze profiles therein are prescribed, which may lead to inconsistency. Above all, the chemical networks implemented in current models are largely limited to the CHNO system with reaction rates measured in the 1980s (e.g., [13]). Many recently updated chemical pathways and rates have not yet been incorporated. This becomes a critical bottleneck in model development and urges timely updates.

### 3.2 Laboratory experiments

Laboratory experiments provide the foundational data and the mechanistic insights in understanding chemical and microphysical processes in reducing atmospheres, whose results are usually fed into numerical models to advance our understanding (Fig. 2).

**Fig. 2:**
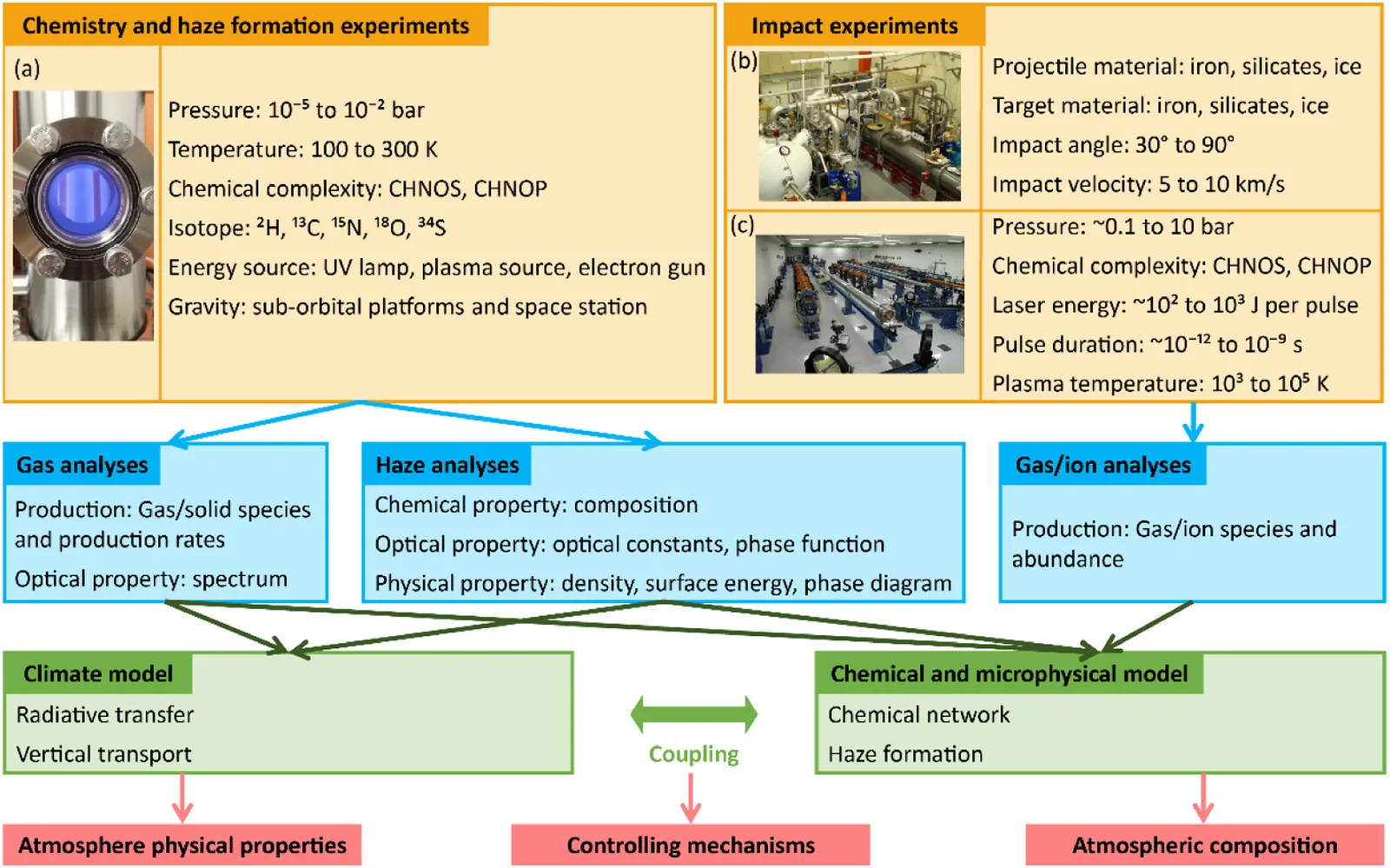
**Schematic overview of the laboratory and simulation studies necessary for the study of reducing atmospheres.** The PHAZER chamber image (a) is taken from https://hub.jhu.edu/2018/12/17/atmospheric-oxygen-does-not-mean-life. The Experimental Impact Laboratory image (b) is taken from https://ares.jsc.nasa.gov/research/laboratories/experimental-impact.html. The Prague Asterix Laser System (PALS) image (c) is taken from https://www.fzu.cz/en/services/equipment-and-technologies/experimental-equipments/pals-prague-asterix-laser-system.

Driven by the space exploration of potentially habitable icy worlds, studies have been conducted on relevant organic chemical networks and haze microphysics across diverse atmospheric conditions. Continuous-flow photochemical chambers, e.g., the Planetary HAZE Research (PHAZER) [15], provide haze particle production rates, gas-phase product yields, particle size distributions, and their chemical and physical properties, which are typically used to set aerosol production rates, gas/solid reaction rates, and haze microphysical parameters in numerical models. Optical analyses, e.g., vacuum Fourier-Transform Infrared Spectroscopy [16], constrain optical properties of the haze particles, which are then adapted to the radiative transfer schemes in simulations. These efforts have successfully reproduced and explained key observations, including vertical extinction profiles and spectral geometric albedos of Titan’s and Pluto’s atmospheres [6,7]. Applications of these advancements to the early Earth have also advanced our understanding (e.g., [13]), while systematic biases exist due to differences in the atmospheric compositions. For example, even at the same temperature, the haze production rates can span two orders of magnitude across the H_2_-rich and CO_2_-rich atmospheres [17,18]. Titan haze analogs produced under the comparable conditions could exhibit order‑unity differences in absorption strength and ∼10% differences in refractive indices over 0.4–3.5 µm, even though individual measurements typically have uncertainties of only ∼4% in the real part and up to ∼10% in the imaginary part [16].

Besides the known variabilities and uncertainties, most currently available experiments are usually limited to the CHNO system (e.g., [13]). Two elements critical to life, sulfur (S) and phosphorus (P), have often been excluded from experimental studies due to their contamination of the experiment platforms and the poisoning concerns. This may be acceptable for highly reducing icy worlds, e.g., Titan and Pluto, but the applicability to the early Earth is uncertain. A recent work with a CHNOS system demonstrated significant differences in haze production and properties when S is present [12], underscoring the need for experiments under more realistic early-Earth-like conditions.

### 3.3 Impact experiments

The recent emergence of advanced large-scale facilities has enabled high-velocity impact experiments and advanced our understanding of impact dynamics, which typically includes light-gas guns [19] and high-power laser systems [20]. These experiments could facilitate transient gas and ion reactions under high‑energy conditions, providing bases for the parameterization of impact‑driven chemistry. However, such efforts are currently limited due to technical challenges in capturing and detecting volatile species at high temporal resolution, which impedes detailed simulations of the atmospheric evolution immediately following impacts. As a consequence, current models often treat impact outcomes as instantaneous injections of gas species [3], which suggests a critical need for quantitative, or at least qualitative, laboratory measurements.

## 4 Solutions

To address the aforementioned challenges, we propose to develop and advance laboratory experiments focusing on the physical and chemical processes in chemically reducing atmospheres, which include studies on photochemical reactions, haze formation, optical and physical property measurements, high-velocity impacts, and related numerical modeling (Fig. 2).

To bridge the knowledge gaps regarding the roles of sulfur and phosphorus in prebiotic atmospheres, investigations involving CHNOS and CHNOP systems are of great importance (Fig. 2). In addition to typical gases such as CH₄, N₂, H₂, and CO₂ used in CHNO systems, introducing H₂S and P/PH_3_ is recommended. Real-time characterization of reaction products and intermediates via synchrotron-based mass spectrometry will help to identify and quantify chemical pathways and rates. This approach will also allow isotopic fractionation analysis (e.g., ¹³C, ²H, ¹⁵N, ¹⁸O, and ³⁴S, if introduced in the reactants; Fig. 2), which is essential for interpreting the atmospheric evolution through isotopes in geological records. Haze particles, expected to form during experiments, should be collected over extended durations for detailed compositional, optical, and physical analyses (see below).

Given the diversity of planetary atmospheres, we recommend systematic haze formation experiments across a range of physical parameters. Pressure conditions should span from 10⁻⁵ to 10⁻² bar, covering haze formation pressure levels on Pluto and those expected on the early Earth (Fig. 2). Moreover, as photochemical hazes on Pluto and Triton appear to form at ∼10^−6^ bar where eddy-diffusion likely differs by orders of magnitude [6], we recommend developing experimental capabilities that can probe products under this pressure, though this may lead to long reaction time and difficult particle detection. Similarly, temperatures are recommended to range from 100 to 300 K, corresponding to the haze-forming regions on Titan and the early Earth, respectively (Fig. 2). Reaching Pluto-like temperatures below 50 K is technically demanding and has not yet been demonstrated, which could be regarded as a longer-term but highly desirable goal. Multiple energy sources, including UV lamps, plasma sources, and electron guns, should be incorporated to simulate the effects of solar photons and energetic particles (Fig. 2), whose roles are currently underexplored. Moreover, experiments with multi-wavelength UV and multi-source particles to simulate different stellar environments would support future exoplanet studies. The influence of gravity on haze microphysics should also be examined to extend our understanding of haze particle aggregation and settling under low gravity (Fig. 2). These micro-gravity experiments are likely to be long-term, resource-intensive projects, but targeted campaigns on sub-orbital platforms or space stations would provide valuable information. With the production of these haze analogs, optical property measurements across UV to IR should be carried out in the short term with existing laboratory setups, such as spectrometers and ellipsometry. In the long term, integrating optical measurements with physical property analyses, including density, surface energy, hygroscopicity, etc., and uncertainty quantification through repeated runs, instrument calibration, and cross-laboratory comparison studies is expected to reduce systematic biases.

High-energy experiments are essential to constrain one of the largest uncertainties in the early planetary atmospheres (Fig. 2). Impact experiments with velocities on the order of ∼10 km/s, achievable with light-gas guns, should be used to simulate frequent impact events in the early Solar System. A number of impact angles should also be considered. Projectile and target materials should include iron, silicates, and ice to mimic the conditions on terrestrial planetary surfaces. Passive spectroscopy can serve as a first attempt to detect the gases and their abundances produced during impacts, as the impact process usually occurs over short timescales with extremely high energies. Nevertheless, monitoring impact-induced chemical dynamics of gases within the reaction chamber remains challenging and warrants further exploration. In addition to the projectile-based impacts, high-energy pulsed-laser experiments that generate laser-induced dielectric breakdown plumes can be a complementary proxy. By systematically varying laser pulse energy (∼10^2^–10^3^ J), pulse duration (ps-ns), gas composition (e.g., N_2_—CO_2_—CH_4_-X), and pressure (∼0.1–10 bar), these experiments can map the yields of key organic products, and quantify their production (e.g., [20]).

Advances in numerical modeling will complement experimental efforts in improving our understanding of chemical and physical processes in reducing atmospheres (Fig. 2). Newly measured reaction networks and haze formation mechanisms should be integrated into existing models to evaluate their influences. Updated optical and physical properties of the reaction products are expected to enhance model databases. Additional processes revealed through experiments should also be incorporated. Above all, the quantified experimental uncertainties should be propagated into photochemical and climate models through systematic sensitivity analyses so that their impact on the derived climate states can be evaluated. Finally, coupling atmospheric chemistry with climate models is necessary to achieve a comprehensive view of the planetary atmospheres. A fully-coupled 3D chemistry-climate model would enable resolution of the interactions among dynamics, radiative transfer, and chemistry.

## CRediT authorship contribution statement

**Lixiang Gu:** Writing – original draft. **Siteng Fan:** Writing – review & editing.

## Declaration of competing interest

The authors declare that they have no conflicts of interest in this work.
